# Lifestyle and Dietary Determinants of Serum Apolipoprotein A1 and Apolipoprotein B Concentrations: Cross-Sectional Analyses within a Swedish Cohort of 24,984 Individuals

**DOI:** 10.3390/nu9030211

**Published:** 2017-02-28

**Authors:** Kasper Frondelius, Madelene Borg, Ulrika Ericson, Yan Borné, Olle Melander, Emily Sonestedt

**Affiliations:** 1 Diabetes and Cardiovascular Disease—Genetic Epidemiology, Department of Clinical Sciences Malmö, Lund University, Jan Waldenströms gata 35, SE-20502 Malmö, Sweden; gbi10kfr@student.lu.se (K.F.); madelene.borg.342@student.lu.se (M.B.); ulrika.ericson@med.lu.se (U.E.); 2 Cardiovascular Epidemiology, Department of Clinical Sciences Malmö, Lund University, Jan Waldenströms gata 35, SE-20502 Malmö, Sweden; yan.borne@med.lu.se; 3 Hypertension and Cardiovascular Disease, Department of Clinical Sciences Malmö, Lund University, Jan Waldenströms gata 35, SE-20502 Malmö, Sweden; olle.melander@med.lu.se; 4 Nutritional Epidemiology, Department of Clinical Sciences Malmö, Lund University, Jan Waldenströms gata 35, SE-20502 Malmö, Sweden

**Keywords:** diet, nutrition, apolipoproteins, epidemiology

## Abstract

Low serum apolipoprotein (Apo) A1 concentrations and high serum ApoB concentrations may be better markers of the risk of cardiovascular disease than high-density lipoprotein (HDL) and low-density lipoprotein (LDL). However, the associations between modifiable lifestyle factors and Apo concentrations have not been investigated in detail. Therefore, this study investigated the associations between Apo concentrations and education, lifestyle factors and dietary intake (macronutrients and 34 food groups). These cross-sectional associations were examined among 24,984 individuals in a Swedish population-based cohort. Baseline examinations of the cohort were conducted between 1991 and 1996. Dietary intake was assessed using a modified diet history method. The main determinants of high ApoA1 concentrations (*r* between 0.05 and 0.25) were high alcohol consumption, high physical activity, non-smoking, and a low body mass index (BMI), and the main determinants of high ApoB concentrations were smoking and a high BMI. The intake of sucrose and food products containing added sugar (such as pastries, sweets, chocolate, jam/sugar and sugar-sweetened beverages) was negatively correlated with ApoA1 concentrations and positively correlated with ApoB concentrations and the ApoB/ApoA1 ratio, whereas the intake of fermented dairy products, such as fermented milk and cheese, was positively correlated with ApoA1 concentrations and negatively correlated with the ApoB/ApoA1 ratio. These results indicate that smoking, obesity, low physical activity, low alcohol consumption and a diet high in sugar and low in fermented dairy products are correlated with an unfavorable Apo profile.

## 1. Introduction

Cardiovascular diseases (CVD) comprise a group of diseases targeting the circulatory system and are considered the most frequent cause of death in the Western world [1]. One of the main causes of CVD is atherosclerotic plaque accumulation in blood vessels, which results in ischemia, inflammation and thrombi, phenomena that can lead to hypoxia-inducing occlusions [2]. Cholesterol participates in the development of atherosclerosis and is thus an important risk factor for CVD [3]. Lipoproteins are the primary transporters of lipids (i.e., cholesterol and free fatty acids) in the body, and the following five different types of lipoproteins are present in the system: very low-, low-, intermediate- and high-density lipoproteins (VLDL, LDL, IDL, HDL, respectively) and chylomicrons. High LDL concentrations, low HDL concentrations, and in particular, a low HDL/LDL ratio, are established markers of the risk of CVD, especially coronary artery disease. The surface of the lipoprotein is equipped with apolipoproteins (Apo), which both guide lipid transportation and interact with specific receptors to facilitate the uptake and deposition of lipids into tissue; thus, they play a central role in cholesterol metabolism [4,5]. ApoA1 represents the major HDL Apo and is the main acceptor of cholesterol when HDL transports cholesterol from the tissues to the liver to be excreted from the body. ApoB, a common denominator for the two forms of ApoB, i.e., ApoB_48_, the truncated form of ApoB; and ApoB_100_, the full form of Apo, is the major Apo of VLDL, IDL, LDL and lipoprotein (a). These molecules transport triacylglycerol (TG) and cholesterol from the liver to cells. Therefore, ApoB concentrations are a measure of the total numbers of all atherogenic particles, including VLDL, IDL and LDL, while ApoA1 is a measure of HDL particles. The results of several epidemiologic studies and clinical trials indicate that high ApoB concentrations, low ApoA1 concentrations and the ApoB/ApoA1 ratio may be better markers of the risk of CVD than LDL, HDL and the LDL/HDL ratio [6,7,8,9]. Several lifestyle and dietary factors play important roles in CVD prevention [10]. The associations between these factors and Apo serum concentrations have been examined in epidemiological studies [11,12,13]. However, there remains a lack of larger studies investigating these factors simultaneously. In particular, there is a lack of studies investigating multiple dietary factors. Thus, this study aimed to examine whether lifestyle and dietary factors (both macronutrients and groups of foods and beverages) are associated with serum Apo parameters (ApoA1 and ApoB concentrations and the ApoB/ApoA1 ratio) in a large population-based cohort of nearly 25,000 individuals. To our knowledge, the association between dietary factors and Apo concentrations has not yet been investigated in a cohort of this size. 

## 2. Materials and Methods

### 2.1. Study Population and Data Collection

The Malmö Diet and Cancer (MDC) study is a population-based cohort of men and women aged 44–74 years. The study’s baseline examinations were conducted between 1991 and 1996 in southern Sweden [14,15,16]. A total of 74,318 individuals living in the city of Malmö were invited to participate in the study through both passive and active recruitment methods. Individuals with inadequate Swedish language skills or a mental disability were excluded from participating in the study. Complete sets of data were collected for 28,098 participants (11,063 males and 17,035 females). The Ethics Committee at Lund University approved the MDC study, and all participants provided informed consent to participate in the study upon entering it. Among those who participated between 1991 and 1994, 50% (*n* = 6103) were invited for an additional visit (after a mean of 0.7 years), during which blood was drawn after an overnight fast for measurements of total cholesterol, HDL-cholesterol (HDL-C) and triglyceride concentrations [17]. LDL-cholesterol (LDL-C) concentrations were calculated using the Friedewald formula, LDL-C = total cholesterol—HDL-C—(triglycerides/2.2), among participants with triglyceride concentrations below 4.0 mmol/L.

The baseline examination comprised a self-administered questionnaire (which was administered to collect data on physical activity, smoking habits, alcohol consumption habits and socioeconomic factors), anthropometric measurements, a dietary assessment and the collection of non-fasting blood samples. Blood components were then separated, frozen and stored in a biobank [18]. ApoA1 and ApoB concentrations were measured by Quest Diagnostics (San Juan Capistraon, CA, USA) in serum samples that had been stored at −80 °C until the analysis was performed in 2013 using an immunonephelometric assay that was run on a Siemens BNII (Siemens, Newark, DE, USA). 

In the present study, we excluded participants with a known history of myocardial infarction or stroke (*n* = 855), as well as individuals with a self-reported diagnosis of diabetes or glucose-lowering medication (*n* = 870) or lipid-lowering medication use (*n* = 907), resulting in a cohort comprising 25,839 individuals. Of these individuals, 613 had no data pertaining to ApoB and ApoA1 serum concentrations or had missing information pertaining to education (*n* = 59), smoking (*n* = 10), leisure-time physical activity (*n* = 171), or body mass index (BMI) (*n* = 30). Thus, the study sample ultimately included 24,984 individuals.

### 2.2. Dietary Variables

Dietary intake was measured using a modified diet history method developed for the MDC study [19]. The method consists of a 168-item semi-quantitative diet history questionnaire designed to collect data spanning a year, as well as a 7-day food record. The dietary questionnaire covered food items that participants frequently consumed during the past year. Participants entered the frequencies at which they consumed specific foods and estimated the sizes of the portions that they ate using a booklet. The 7-day food record included information covering cooked lunches and dinners, cold beverages (including alcoholic beverages) and dietary supplements. Information regarding medications and natural remedies was also recorded. These measures were complemented by a 45–60 min diet history interview on food choices, portion sizes and food preparation practices that was performed by trained professionals. Participants who entered the study after 1 September 1994, underwent a less thorough interview than those who entered prior to that date (45 minutes as opposed to 1 h). To account for this difference, we adjusted for participant screening dates in the analyses. Average daily food intake (g/day) was calculated by combining data from the interview, the 7-day food record and the questionnaire covering the past year. The data were converted into information on nutrient intake via the MDC food and nutrient database, which was developed from PC-kost2-93 of the Swedish National Food Agency.

In this study, we examined both macronutrients and foods. The following macronutrients (expressed as E% of non-alcohol energy intake) were examined: total fat, saturated fat, monounsaturated fat, polyunsaturated fat, omega-3 and omega-6 polyunsaturated fatty acids, protein, total carbohydrates, sucrose, and fiber (g/MJ). The foods (expressed as g/MJ of total energy intake) were categorized into 34 groups to cover the entire diet and, in particular, to separate foods based on sugar content (Table S1). The intakes were divided into decentiles (if the majority of individuals consumed the specific food) or four groups (if fewer than 50% of individuals consumed the food), with zero-consumers categorized into one group. Because of differences in the density of soft and hard bread, we multiplied intake of hard bread by 1.667 before constructing the high-fiber and low-fiber bread groups. The validity and reproducibility of the dietary assessment method are presented elsewhere [20,21,22].

### 2.3. Other Variables

The relationships between the following variables and serum Apo concentrations were examined. The participants were categorized into the following six groups according to age, with each age group spanning 5 years: 45–50, 50–55, 55–60, 60–65, 65–70 and 70–75. We estimated alcohol habits by combining information from the questionnaire and the 7-day record. The participants who indicated that they did not consume alcohol in the last year in the questionnaire and in the 7-day record were coded as non-consumers. The remaining subjects were divided into sex-specific quintiles based on their intake during the 7-day period in which they kept a food record. The groups were categorized using the following cut off values: 3.4, 9.1, 15.7, 25.7 g/day (men) and 0.9, 4.3, 8.1, 14.0 g/day (women). According to BMI, which was calculated based on measured weight and height and expressed as kg/m^2^, the participants were categorized into the following groups: underweight (BMI < 18.5), normal weight (BMI 18.5–25), overweight (BMI 25–30), obese (BMI 30–35) and severely obese (BMI > 35). Physical activity was measured through the self-reported questionnaire, in which participants reported their performance of 17 types of leisure-time physical activities in minutes per week and per season. Each activity was assigned a factor based on its intensity and multiplied by the time that an individual spent performing it. A total score accounting for the time, frequency and intensity of the activities was created for each participant. This score was then divided into quintiles. We categorized participants into the following three categories based on their smoking habits: current smokers (including irregular smokers), never-smokers and ex-smokers. Participants were also classified into the following five education status groups based on the highest level of education that they had completed: elementary school, primary and secondary school, upper secondary school, further education without a degree, and university degree.

### 2.4. Statistical Analyses

Statistical analyses were conducted using SPSS Statistics (version 22; IBM Corporation, Armonk, NY, USA). All analyses were performed separately for men and women. The ApoA1 and ApoB concentrations and the ApoB/ApoA1 ratios were normally distributed. Differences in age-adjusted mean serum Apo concentrations according to lifestyle factors were tested using general linear model. First, crude correlation analyses (Pearson and Spearman’s rho) were used to examine the associations between food intake and Apo concentrations. The results of these analyses showed that the correlation coefficients were very similar across the tests. Then, partial correlations between lifestyle factors and Apo concentrations were computed. In the basic model, we adjusted for age and screening date. For dietary variables (macronutrients and food groups), we also adjusted for total energy intake. In the full multivariable model, we also included BMI, leisure-time physical activity, alcohol consumption habits, education level, and smoking habits as covariates. Then, we used stepwise linear regression analyses to mutually adjust for all lifestyle and nutrient factors simultaneously. The adjustments performed in the model were similar to those performed in the full multivariable model. We used a *p*-value < 0.05 as an entry criterion and a *p*-value > 0.10 as a removal criterion. A similar analytic strategy was used to mutually adjust for all food groups simultaneously. Variance in Apo concentrations according to lifestyle factors and food intake was also determined. These variances were defined as *r*^2^ and expressed as percentage of total variance. For the sensitivity analysis, we eliminated participants who had changed their diet substantially in the past [23,24] or were likely to misreport their energy intake. Energy misreporters were identified by comparing quotient of the reported total energy intake divided by the basal metabolic rate (BMR) with the estimated physical activity level (based on self-reports of physical activity at work, during leisure-time, and during household work, as well as estimated sleep durations, self-care time durations and passive time durations), which was expressed as the total energy expenditure divided by the BMR [25].

Statistical significance was set at *p* < 0.05, with 2-sided *p*-values. For the correlation analyses including lifestyle factors and macronutrients, we also indicated correlations *p* < 0.001. When analyzing the 34 food groups, we used Bonferroni to correct for multiple testing. Significance was defined as a *p*-value less than 0.0015 (0.05/34 categories). Nominal significance was defined as a p-value between 0.0015 and 0.05.

## 3. Results

### 3.1. Lifestyle Determinants of Apo Concentrations

The study sample consisted of 15,621 (63%) women and 9363 (37%) men. The mean ApoA1 concentration was 1.64 (SD: 0.28) g/L for women and 1.46 (SD: 0.25) g/L for men. The mean ApoB concentration was 1.04 (SD: 0.26) g/L for women and 1.10 (SD: 0.25) g/L for men. The mean ApoB/ApoA1 ratio was 0.65 (SD: 0.20) for women and 0.78 (SD: 0.22) for men. We observed strong correlations between HDL-C and ApoA1 (*r* = 0.72) and between LDL-C and ApoB (*r* = 0.74) in the subsample (*n* = 4653) of individuals with measurements of these markers. Age-adjusted mean Apo concentrations according to different lifestyle factor categories are presented in Table 1. The results of our analyses showed that after adjustment for age and the date that blood samples were collected, ApoA1 concentrations were, on average, 13% lower in men than in women; ApoB concentrations were 4% higher in men than in women; and ApoB/ApoA1 ratios were 18% higher in men than in women.

The partial correlations between Apo concentrations and lifestyle and dietary factors are presented in Table 2 using three different models. Lower correlations were generally observed in the full multivariable adjusted model than in the basic model. We hereafter present results from the full multivariable model controlling for several potential confounders. Among the examined lifestyle and dietary factors, higher alcohol consumption and lower BMI were the main determinants of ApoA1 concentrations; a higher BMI was the main determinant of ApoB concentrations; and a higher BMI, smoking, and lower alcohol consumption were the main determinants of the ApoB/ApoA1 ratio. The correlation between alcohol consumption and ApoA1 concentrations (*r* = 0.25 in men; *r* = 0.19 in women) was the strongest correlation observed between the two sets of parameters. Men and women in the group with the highest alcohol consumption (cut off values: 25.7 g/day for men and 14.0 g/day for women) had ApoA1 concentrations that were 16% and 11% higher, respectively, than those of men and women in the non-consumer group (Table 1). BMI was moderately negatively correlated with ApoA1 concentrations (*r* = −0.19 for men and −0.18 for women) and positively correlated with ApoB concentrations (*r* = 0.18 for men and women) and the ApoB/ApoA1 ratio (*r* = 0.25 for men and *r* = 0.24 for women). Severely obese (BMI > 35) men and women displayed ApoB/A1 ratios that were 46% and 32% higher than those displayed by underweight (BMI < 18.5) individuals. Physical activity was weakly but positively correlated with ApoA1 concentrations (*r* = 0.06 in men and *r* = 0.05 in women) and was negatively correlated with ApoB concentrations in men (*r* = 0.024). Physical activity was also negatively correlated with the ApoB/ApoA1 ratio (*r* = 0.05 in men and 0.03 in women). Smoking was weakly but significantly negatively correlated with ApoA1 concentrations and was positively correlated with ApoB concentrations and the ApoB/ApoA1 ratio. The highest ApoB concentrations and the highest ApoB/ApoA1 ratio were observed among smokers (Table 1). 

### 3.2. Macronutrient Determinants of Apo Concentrations

Among the investigated macronutrients, sucrose was moderately correlated with ApoA1 concentrations (*r* = −0.09 in men and r = −0.06 in women), ApoB concentrations in women (*r* = 0.05) and the ApoB/ApoA1 ratio (*r* = 0.06 in men and *r* = 0.07 in women). Fat intake was correlated mainly with ApoA1 concentrations (*r* = 0.08 in men and *r* = 0.07 in women), as well as with ApoB concentrations (significant only in men), and was negatively correlated with the ApoB/ApoA1 ratio. Regarding the correlations between the types of fat and the above parameters, the correlation between MUFA and ApoA1 was the strongest correlation observed in men (*r* = 0.08) and the correlation between SFA and ApoA1 was the strongest correlation observed in women (*r* = 0.07) (Table 2). 

### 3.3. Food Group Determinants of Apo Concentrations

**Figure 1:**
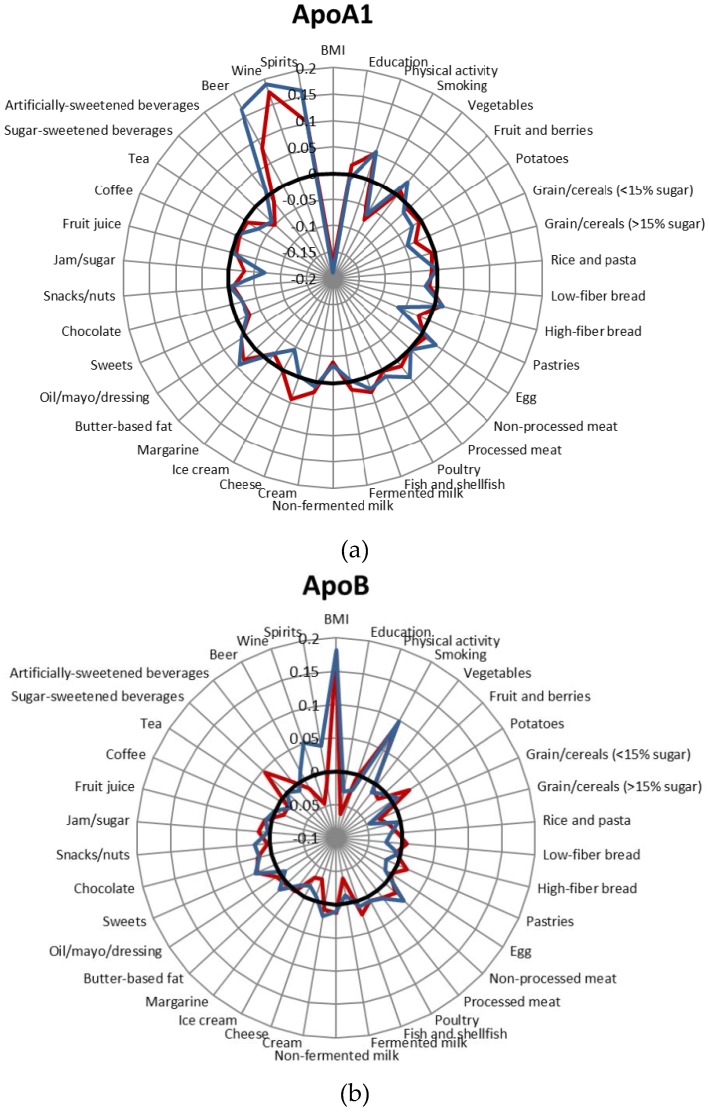

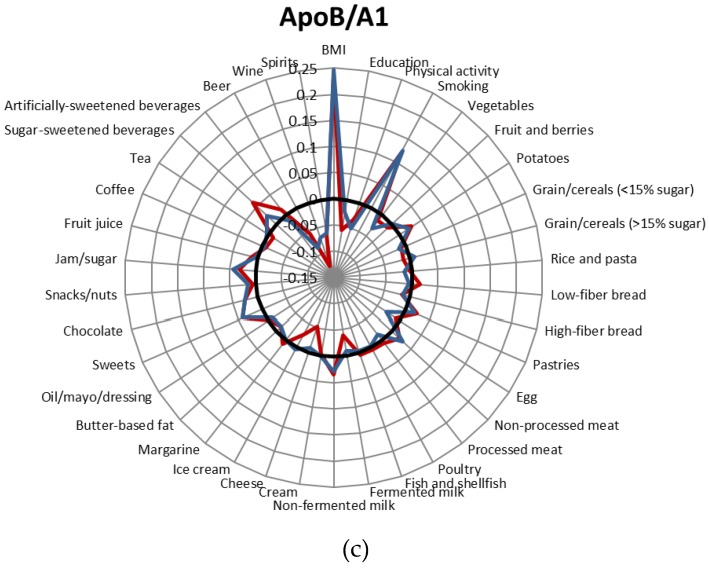
Correlations between lifestyle factors and food intake and ApoA1 concentrations (**a**), ApoB concentrations (**b**) and the ApoB/ApoA1 ratio (**c**). Blue: men; red: women; black: *r* = 0. Adjusted for age, screening date, total energy intake, BMI, leisure-time physical activity, alcohol and smoking habits. The analyses including beer, wine and spirits were not adjusted for alcohol.

The correlations between the 34 groups of foods and beverages and Apo concentrations are presented in Figure 1, Tables S2 and S3. When we performed the sensitivity analysis excluding individuals who reported substantial changes in their food habits in the past and potentially misreported energy, the correlations were somewhat similar. Therefore, we reported the results of the entire study sample. The correlations between alcoholic beverages (wine, beer, spirits) and Apo concentrations were the strongest correlations observed in the full multivariable model adjusting for potential lifestyle confounders (Figure 1). Wine was more strongly correlated with ApoA1 (especially in women) than the other alcoholic beverages. In addition, the intake of products containing added sugar such as pastries, sweets, chocolate, jam/sugar and sugar-sweetened beverages was relatively strongly correlated with Apo concentrations compared with intakes of products in other food groups. High intake of these products was associated with lower ApoA1 concentrations but higher ApoB concentrations and a higher ApoB/ApoA1 ratio. The correlations between jam/sugar and ApoA1 concentrations among men (*r* = −0.07), between sugar-sweetened beverages and the ApoB/ApoA1 ratio among women (*r* = 0.06), and between pastries and the ApoA1 among men (*r* = 0.06) were the strongest correlations observed in the analyses. Nearly all of the correlations between Apo and sweets, jam/sugar and sugar-sweetened beverages remained significant in the mutually adjusted model in which all food groups were included simultaneously.

Several dairy products were significantly correlated with Apo concentrations, especially in women. Non-fermented milk was significantly inversely correlated with ApoA1 concentrations and positively correlated with the ApoB/ApoA1 ratio in both men and women. In women, fermented dairy products, such as fermented milk and cheese, showed the opposite association with ApoB concentrations and the ApoB/ApoA1 ratio, i.e., they were significantly inversely correlated with ApoB concentrations and the ApoB/ApoA1 ratio. These correlations remained significant in the mutually adjusted model in which all food groups were included simultaneously. 

The other food group determinants of Apo parameter concentrations and ratios (i.e., those that reached the Bonferroni-corrected significance level and remained at least nominally significant in the mutually adjusted model) were as follows: high ApoA1 = high intake of fish (women), butter-based fat (women and men), eggs (men), and processed meat (men) and low intake of low-sugar grains/cereals (women and men) and ice cream (men); high ApoB = high intake of potato (women) and non-processed meat (men) and low intake of low-sugar grains (women and men) and ice cream (women); and the ApoB/A1 ratio = high intake of potato (women) and low intake of ice cream (women).

The combination of lifestyle and food variables explained only a minor percentage of the variances in Apo concentrations. These factors explained similar percentages of the variances in ApoA1 concentrations in men (11.6%) and women (9.2%). However, regarding ApoB concentrations and the ApoB/A1 ratio, the above factors explained more of the variances in these parameters among women (10.7% and 13.5%, respectively) than among men (5.4% and 9.2%, respectively). 

We also examined the correlations between lifestyle and dietary factors and HDL-C concentrations, LDL-C concentrations and the LDL-C/HDL-C ratio in the subsample (*n* = 4653) of individuals with measurements of those markers (Table S4). The combination of the lifestyle and food variables explained more of the variance in HDL-C concentrations (men: 17.7%; women: 15.1%) than in ApoA1 concentration, less of the variances in LDL-C concentrations (men: 3.5%; women 6.2%) than in ApoB concentrations, and similar percentages of the variances in the LDL-C/HDL-C ratio (men: 9.7%; women: 13.4%) than in the ApoB/ApoA1 ratio.

## 4. Discussion

### 4.1. Main Findings of the Study

We identified several lifestyle and dietary determinants of the Apo concentrations in this cross-sectional study comprising 24,984 individuals. High alcohol consumption and a low BMI were the main determinants of higher ApoA1 concentrations; a high BMI was the main determinant of higher ApoB concentrations; and a high BMI, smoking, and low alcohol consumption were the main determinants of a higher ApoB/ApoA1 ratio. Regarding the food groups, high intakes of sugary foods and low intakes of fermented dairy products, such as cheese and fermented milk, were the determinants of higher ApoB concentrations and a higher ApoB/ApoA1 ratio and lower ApoA1 concentrations.

### 4.2. Comparisons with Other Studies

In the present study, we observed a dose–response relationship between alcohol consumption and all Apo concentrations. Specifically, we noted that the highest ApoA1 concentrations and lowest ApoB concentrations and ApoB/ApoA1 ratios were displayed by those with the highest levels of alcohol consumption. Moderate amounts of alcohol intake (≈20 g/day for men and ≈10 g/day for women) have been shown to associate with protection against CVD [26]. Only the highest quintiles (>25.7 g alcohol/day for men and >14.0 g alcohol/day for women) of participants reported a moderate or high alcohol intake. Wine was more strongly associated with Apo concentrations (particularly ApoA1 concentrations in women) than the other alcoholic beverages. However, the above association was not stronger than that between total alcohol intake and Apo concentrations, indicating that alcohol content is mainly responsible for the effects of alcoholic beverages on Apo concentrations. High alcohol intake was correlated with higher ApoA1 concentrations both in a study examining 1566 subjects [13] and in a middle-aged Chinese population (*n* = 10,154) [12]. Similarly, in a previous Swedish population-based study including 2907 individuals (25–74 years), alcohol was inversely correlated with the ApoB/ApoA1 ratio. The study also noted stronger associations between alcohol and the ApoB/ApoA1 ratio in women and showed that the associations were mainly attributable to wine consumption [11]. Although we did not observe any detrimental associations between high alcohol intake and Apo concentrations, the negative aspects of high alcohol consumption are well known [27], and we advised caution when assessing the risks versus benefits of alcohol consumption.

High BMI and smoking were correlated with lower ApoA1 concentrations and higher ApoB concentrations and a higher ApoB/ApoA1 ratio. These results are consistent with those of previous studies [11,28]. A higher education level is often associated with a healthier lifestyle and a lower risk of developing a disease than a lower education level [29]. In our study, the correlation between education and Apo concentrations was weakened after adjustment for other lifestyle variables, suggesting that education has a small independent association on Apo concentrations. 

Regarding macronutrients, low sucrose intake was the main dietary determinant of higher ApoA1 concentrations and a lower ApoB/ApoA1 ratio in both genders. In addition, the food items containing added sugar, such as sweets, jam/sugar, chocolate, sugar-sweetened beverages and pastries, were the most strongly associated with unfavorable Apo concentrations. Observational studies have shown that higher intake of added sugar is associated with an increased incidence of CVD and CVD-mortality [30,31] and that sugar consumption seems to adversely affect blood lipid concentrations, resulting in a more atherogenic profile [11,32,33,34]. We previously showed that sugars and sugar-rich foods were most strongly correlated with fasting serum HDL-C concentrations in the MDC cohort [32,35,36]. Fat intake was positively correlated with ApoA1 and ApoB concentrations (significant only in men) and negatively correlated with the ApoB/ApoA1 ratio. These results support using the ApoB/ApoA1 ratio in combination with other variables (such as the LDL/HDL or blood pressure) to determine the risk for CVD. Different dairy products displayed different correlations with Apo concentrations. Lactic acid-fermented dairy products, including cheese and fermented milk, were associated with favorable Apo profile. In contrast, non-fermented milk was associated with unfavorable Apo profile. Evidence from animal and human studies suggests that fermented dairy products lower overall cholesterol levels, possibly because such products contain a large amount of bacteria [37]. Although the correlations between Apo concentrations and food intake were relatively low, the results of the above analysis indicate that specific diet intervention studies should be conducted to investigate the impact of dietary intake on Apo concentrations further.

### 4.3. Strengths and Limitations

This study was based on observational cross-sectional data. We excluded users of lipid-lowering medication (3% of the study population) and therefore have limited the bias resulting from participants’ awareness of their lipoprotein concentrations. Due to the large number of participants, the study had strong power to observe even moderate correlations between lifestyle factors and Apo concentrations. Exposures, including both lifestyle factors and dietary intake, are frequently misreported in epidemiologic studies. Therefore, it is important to keep in mind that the lower correlations between dietary factors and Apo concentrations observed herein may, to some extent, be attributable to the fact that habitual food intake is affected by a higher degree of measurement error than for other lifestyle factors. For example, BMI and smoking may be more easily and accurately reported than habitual food intake. The methods used to collect dietary information were specifically developed to capture the habitual diets of the participants enrolled in the MDC study, and the relative validity of this method is high compared to that of other methods. In addition, we investigated the composition of nutrients and foods (E% and g/MJ), adjusted for total energy intake, and excluded potential misreporters from sensitivity analyses to reduce the impact of measurement errors. 

Apo concentrations were measured in non-fasting blood samples. Lipid and lipoprotein concentrations are traditionally measured in fasting blood samples; however, triglyceride concentrations seem to be a better predictor of risk when measured in non-fasting blood samples [38,39,40]. Fasting and non-fasting lipid, lipoprotein and Apo levels seem to be valuable for risk prediction [41,42]. Compared to measuring HDL-C and LDL-C, Apo assays are expensive and not widely available in the clinic. The advantages of using Apo concentrations is that they can be measured in non-fasting samples [43] and that ApoB, compared to LDL-C, is a better indicator of the total number of atherogenic particles.

## 5. Conclusions

Apo concentrations may be better risk markers for CVD than LDL and HDL; therefore, it is important to identify the dietary determinants of Apo concentrations. The current results indicated that smoking, obesity, low alcohol consumption and a diet high in sugar and low in fermented dairy products were associated with an unfavorable apolipoprotein profile. However, these factors explained only a minor percentage (5%–14%) of the variance in apolipoprotein concentrations noted herein. Future studies, including both large and well-powered cohort studies, as well as diet intervention studies, should investigate the impact of dietary intake on Apo concentrations.

## Figures and Tables

**Table 1:** Mean (95% CI) apolipoprotein concentrations according to lifestyle factors in the 9363 men and 15,621 women enrolled in the Malmö Diet and Cancer cohort.

Variables	Men				Women			
	*N*	ApoA1	ApoB	ApoB/A1	*N*	ApoA1	ApoB	ApoB/A1
**All**	9363	1.45 (0.003)	1.10 (0.003)	0.78 (0.002)	15,621	1.66 (0.002)	1.06 (0.002)	0.66 (0.002)
**Age groups**		*p* = 3 × 10^−4^	*p* = 0.02	*p* = 0.08		*p* = 1 × 10^−23^	*p* < 10^−150^	*p* = 6 × 10^−146^
45–50 years	1027	1.45 (0.008)	1.08 (0.008)	0.77 (0.007)	4035	1.62 (0.004)	0.94 (0.004)	0.60 (0.003)
50–55 years	2280	1.44 (0.005)	1.10 (0.005)	0.78 (0.005)	3091	1.64 (0.005)	1.00 (0.004)	0.63 (0.004)
55–60 years	1933	1.45 (0.006)	1.11 (0.006)	0.79 (0.005)	2608	1.66 (0.005)	1.06 (0.005)	0.66 (0.004)
60–65 years	2053	1.47 (0.005)	1.11 (0.006)	0.78 (0.005)	2823	1.66 (0.005)	1.11 (0.005)	0.69 (0.004)
65–70 years	1229	1.46 (0.007)	1.11 (0.007)	0.78 (0.006)	1662	1.65 (0.007)	1.14 (0.006)	0.71 (0.005)
70–75 years	841	1.48 (0.009)	1.11 (0.010)	0.77 (0.008)	1402	1.70 (0.008)	1.16 (0.007)	0.70 (0.006)
**Education level**		*p* = 1 × 10^−4^	3 × 10^−4^	*p* = 4 × 10^−9^		*p* = 3 × 10^−24^	6 × 10^−39^	6 × 10^−55^
Elementary	4188	1.45 (0.004)	1.12 (0.004)	0.79 (0.004)	5982	1.63 (0.004)	1.10 (0.003)	0.69 (0.003)
Primary and secondary	1852	1.47 (0.006)	1.10 (0.006)	0.77 (0.005)	4780	1.67 (0.004)	1.06 (0.004)	0.66 (0.003)
Upper secondary	1133	1.46 (0.007)	1.09 (0.008)	0.77 (0.007)	1110	1.67 (0.008)	1.05 (0.007)	0.65 (0.006)
Further education without a degree	897	1.48 (0.008)	1.09 (0.008)	0.76 (0.007)	1354	1.68 (0.007)	1.04 (0.007)	0.64 (0.005)
University degree	1293	1.47 (0.007)	1.09 (0.007)	0.76 (0.006)	2395	1.69 (0.006)	1.02 (0.005)	0.62 (0.004)
**Physical activity**		9 × 10^−10^	*p* = 2 × 10^−4^	*p* = 2 × 10^−11^		2 × 10^−22^	*p* = 2 × 10^−4^	5 × 10^−17^
Quintile 1	1858	1.43 (0.006)	1.13 (0.006)	0.81 (0.005)	3073	1.62 (0.005)	1.08 (0.004)	0.69 (0.004)
Quintile 2	1878	1.45 (0.006)	1.10 (0.006)	0.78 (0.005)	3142	1.65 (0.005)	1.08 (0.004)	0.67 (0.004)
Quintile 3	1877	1.46 (0.006)	1.11 (0.006)	0.78 (0.005)	3144	1.66 (0.005)	1.06 (0.004)	0.66 (0.004)
Quintile 4	1890	1.47 (0.006)	1.10 (0.006)	0.77 (0.005)	3130	1.67 (0.005)	1.06 (0.004)	0.65 (0.004)
Quintile 5	1860	1.48 (0.006)	1.09 (0.006)	0.75 (0.005)	3132	1.68 (0.005)	1.06 (0.004)	0.65 (0.004)
**BMI groups**		*p* = 4 × 10^−56^	*p* = 8 × 10^−52^	*p* = 1 × 10^−96^		*p* = 6 × 10^−120^	*p* = 3 × 10^-112^	*p* = 1 × 10^−201^
Underweight	52	1.53 (0.034)	0.92 (0.034)	0.61 (0.030)	238	1.72 (0.017)	0.95 (0.016)	0.57 (0.012)
Normal	3575	1.51 (0.004)	1.06 (0.004)	0.72 (0.004)	8239	1.70 (0.003)	1.03 (0.003)	0.62 (0.002)
Overweight	4628	1.44 (0.004)	1.13 (0.004)	0.80 (0.003)	5156	1.62 (0.004)	1.10 (0.003)	0.70 (0.003)
Obese	964	1.39 (0.008)	1.16 (0.008)	0.85 (0.007)	1573	1.57 (0.007)	1.15 (0.006)	0.75 (0.005)
Severely obese	145	1.39 (0.020)	1.21 (0.021)	0.89 (0.018)	418	1.53 (0.013)	1.12 (0.012)	0.75 (0.009)
**Alcohol habits**		*p* = 2 × 10^−123^	*p* = 4 × 10^−6^	*p* = 9 × 10^−19^		*p* = 5 × 10^−155^	2 × 10^−18^	*p* = 1 × 10^−94^
Non-consumers	388	1.34 (0.012)	1.10 (0.013)	0.84 (0.011)	1105	1.57 (0.008)	1.09 (0.007)	0.71 (0.006)
Quintile 1	1750	1.40 (0.006)	1.09 (0.006)	0.80 (0.005)	2826	1.60 (0.005)	1.09 (0.005)	0.70 (0.004)
Quintile 2	1733	1.42 (0.006)	1.09 (0.006)	0.79 (0.005)	2877	1.62 (0.005)	1.08 (0.005)	0.68 (0.004)
Quintile 3	1812	1.46 (0.006)	1.10 (0.006)	0.77 (0.005)	2924	1.65 (0.005)	1.06 (0.005)	0.66 (0.004)
Quintile 4	1826	1.49 (0.006)	1.11 (0.006)	0.76 (0.005)	2937	1.70 (0.005)	1.05 (0.005)	0.64 (0.004)
Quintile 5	1854	1.56 (0.005)	1.13 (0.006)	0.75 (0.005)	2952	1.75 (0.005)	1.05 (0.005)	0.61 (0.004)
**Smoking habits**		*p* = 3 × 10^−5^	*p* = 9 × 10^−19^	4 × 10^−22^		*p* = 3 × 10^−26^	1 × 10^−36^	2 × 10^−59^
Never-smokers	2729	1.46 (0.005)	1.07 (0.005)	0.75 (0.004)	6939	1.66 (0.003)	1.06 (0.003)	0.66 (0.002)
Ex-smokers	3934	1.47 (0.004)	1.11 (0.004)	0.77 (0.004)	4302	1.68 (0.004)	1.04 (0.004)	0.64 (0.003)
Smokers	2700	1.44 (0.005)	1.13 (0.005)	0.81 (0.004)	4380	1.62 (0.004)	1.11 (0.004)	0.71 (0.003)

All values are adjusted for age and screening date. Differences in mean serum apolipoprotein concentrations according to lifestyle factors were tested using a general linear model. Abbreviations: Apo, apolipoprotein; BMI, body mass index.

**Table 2:** Partial correlation between lifestyle and dietary factors and serum apolipoprotein concentrations among the 9363 men and 15,621 women enrolled in the Malmö Diet and Cancer cohort study.

Variables	Basic Multivariable Model ^1^			Full Multivariable Model ^2^			Mutually Adjusted Model ^3^		
	ApoA1	ApoB	ApoB/A1	ApoA1	ApoB	ApoB/A1	ApoA1	ApoB	ApoB/A1
**Men**									
Alcohol habits	0.242 **	0.050 **	−0.097 **	0.253 **	0.041 **	−0.113 **	0.233 **	0.036 **	−0.103 **
BMI	−0.176 **	0.180 **	0.238 **	−0.189 **	0.182 **	0.247 **	−0.194 **	0.174 **	0.240 **
Education	0.046 **	−0.045 **	−0.066 **	−0.008	−0.030 *	−0.023 *	N.S.	−0.032 *	−0.025 *
Physical activity	0.071 **	−0.040 **	−0.073 **	0.055 **	−0.024 *	−0.051 **	0.063 **	−0.017	−0.051 **
Smoking habits	−0.027 *	0.093 **	0.101 **	−0.063 **	0.098 **	0.126 **	−0.065 **	0.094 **	0.125 **
Carbohydrates	−0.109 **	−0.064 **	0.013	−0.087 **	−0.033 *	0.027 *	-	-	-
Sucrose	−0.118 **	−0.007	0.063 **	−0.090 **	−0.009	0.059 **	−0.073 **	0.032 **	0.069 **
Fiber	−0.060	−0.048 **	−0.009	−0.025 *	−0.013	0.002	-	-	-
Protein	0.024 *	−0.054 **	0.031 *	−0.012	0.022	0.014	N.S.	0.038 **	0.039 **
Fat	0.095 **	0.046 **	−0.020 *	0.075 **	0.027 *	−0.025 *	0.055 **	0.042 **	N.S
SFA	0.062 **	−0.036 **	−0.010	0.053 **	0.027 *	−0.013	-	-	-
MUFA	0.097 **	0.054 **	−0.015	0.077 **	0.028 *	−0.025 *	-	-	-
PUFA	0.053 **	0.010	−0.030 *	0.034 **	−0.010	−0.028 *	-	-	-
Omega-3 PUFA	0.082 **	0.039 **	−0.014	0.054 **	0.023 *	−0.011	-	-	-
Omega-6 PUFA	0.035 *	−0.013	−0.031 *	0.019 *	−0.020	−0.027 *	-	-	-
**Women**									
Alcohol habits	0.212 **	−0.074 **	−0.166 **	0.191 **	−0.047 **	−0.138 *	0.176 **	−0.038 **	−0.124 **
BMI	−0.196 **	0.187 **	0.251 **	−0.175 **	0.179 **	0.238 **	−0.180 **	0.179 **	0.237 **
Education	0.079 **	−0.100 **	0.177 **	0.018 *	−0.064 **	−0.059 **	N.S.	−0.061 **	−0.054 **
Physical activity	0.080 **	−0.034 **	−0.069 **	0.051 **	−0.008	−0.034 **	0.055 **	N.S.	−0.034 **
Smoking habits	−0.046 **	0.075 **	0.089 **	−0.075 **	0.092 **	0.120 **	−0.081 **	0.093 **	0.120 **
Carbohydrates	−0.097 **	0.008	0.056 **	−0.084 **	0.016 *	0.057 **	-	-	-
Sucrose	−0.083 **	0.049 **	0.079 **	−0.061 **	0.046 **	0.067 **	−0.018 *	0.045 **	0.049 **
Fiber	0.004	−0.033 **	−0.027 *	−0.009	−0.015 *	−0.009	0.023 **	-	-
Protein	0.042 **	−0.006	−0.023 *	0.031 **	−0.016 *	−0.026 *	0.029 **	N.S.	N.S.
Fat	0.081 **	−0.004	−0.046 *	0.070 **	−0.007	−0.043 **	0.062 **	N.S.	−0.032 **
SFA	0.082 **	−0.015	−0.054 **	0.071 **	−0.011	−0.046 **	-	-	-
MUFA	0.057 **	0.012	−0.021 *	0.051 **	0.003	−0.027 *	-	-	-
PUFA	0.023 *	0.007	−0.007	0.018 *	−0.002	−0.012	-	-	-
Omega-3 PUFA	0.051 **	0.001	−0.026	0.030 **	0.002	−0.016	-	-	-
Omega-6 PUFA	0.013	0.004	−0.005	0.012	−0.005	−0.012	-	-	-

* *p* < 0.05, ** *p* < 0.001. ^1^: Basic model adjusted for age and screening date. Dietary factors were also adjusted for total energy intake. ^2^: Multivariable model adjusted for age, screening date, BMI, leisure-time physical activity, alcohol consumption and smoking habits. Dietary factors were also adjusted for total energy intake. ^3^: Stepwise backward elimination linear regression analysis, in which all lifestyle variables and sucrose, fiber, protein and fat were simultaneously included. The model was adjusted for age, screening data and total energy intake. N.S. indicates an eliminated variable with *p* > 0.10. Abbreviations: Apo, apolipoprotein; BMI, body mass index; SFA, saturated fatty acids; MUFA, monounsaturated fatty acids; PUFA, polyunsaturated fatty acids.

## Supplementary Materials

The following are available online at http://www.mdpi.com/2072-6643/9/3/211/s1: Table S1: Definition of food groups; Table S2: Partial correlation coefficients between dietary intake and serum apolipoprotein concentrations among men in the Malmö Diet and Cancer cohort (1991–1996); Table S3: Partial correlation coefficients between dietary intake and the serum apolipoprotein concentrations among women in the Malmö Diet and Cancer cohort (1991–1996); Table S4: Partial correlation between lifestyle factors, dietary intake and serum HDL-C and LDL-C concentrations among 1844 men and 2809 women in the Malmö Diet and Cancer cohort (1991–1994).
