# Supplementary material for: Lifestyle and Dietary Determinants of Serum Apolipoprotein A1 and Apolipoprotein B Concentrations: Cross-Sectional Analyses within a Swedish Cohort of 24,984 Individuals
Source: Nutrients. 2017 Feb 28;9(3):211. doi: 10.3390/nu9030211 (PMC5372874; doi:10.3390/nu9030211)
Supplement: Supplementary file 1 [file nutrients-09-00211-s001.docx]

Supplementary Materials: Lifestyle and Dietary Determinants of Serum Apolipoprotein A1 and Apolipoprotein B concentrations: Cross-Sectional Analyses within a Swedish Cohort of 24,984 Individuals

Kasper Frondelius, Madelene Borg, Ulrika Ericson, Yan Borné, Olle Melander, Emily Sonestedt

**Table S1.** Definition of food groups

| **Food group** | **Description** |
| --- | --- |
| Vegetables | Vegetables |
| Fruit and berries | Fruit and berries |
| Potatoes | Potato (boiled, fried and deep fried) |
| Grain/Cereals <15% sugar | Flour, grains, cereals <15% sugar |
| Grain/Cereals >15% sugar | Grains, cereals >15% sugar |
| Low-fibre bread | Soft bread <6% fiber, crisp bread <20% fiber |
| High-fibre bread | Soft bread >6% fiber, crisp bread > 20 % fiber |
| Rice and pasta | Rice and pasta |
| Pastries | Biscuits, buns, cakes and pastry |
| Egg | Egg |
| Non-processed meat | Pork, beef, lamb, offal, and game meat |
| Processed meat | Cold cuts of meat (pork, beef, offal), sausage |
| Poultry | Poultry |
| Fish and shellfish | Fish, fish products, and seafood |
| Fermented milk | Yoghurt and sour milk |
| Non-fermented milk | Regular milk (non-fermented) |
| Cream | Cream |
| Cheese | Cheese (including cottage cheese) |
| Ice cream | Ice cream and sherbet |
| Margarine | Margarine |
| Butter-based fat | Butter and milk-based margarine |
| Oil/mayo/dressing | Oil, lard, coconut oil, mayonnaise, dressing |
| Sweets | Sweets |
| Chocolate | Chocolate |
| Snacks/nuts | Popcorn, potato crisps, nuts |
| Jam/Sugar | Jam, marmalade, honey, sugar |
| Fruit juice | Fruit juice (no added sugar) |
| Coffee | Coffee |
| Tea | Tea |
| Sugar-sweetened beverages | Soft drinks and lemonade sweetened with sugar |
| Artificially-sweetened beverages | Soft drinks and lemonade with low-calorie sweeteners |
| Beer | Beer |
| Wine | Wine |
| Spirits | Spirit |

**Table S2.** Partial correlation coefficients between dietary intake and the serum apolipoprotein concentrations among men in the Malmö Diet and Cancer cohort (1991–1996)

|  | **Basic multivariable model^1^** | | | **Full multivariable model^2^** | | | **Mutually adjusted model^3^** | | |
| --- | --- | --- | --- | --- | --- | --- | --- | --- | --- |
| **Food group** | **ApoA1** | **ApoB** | **ApoB/A1** | **ApoA1** | **ApoB** | **ApoB/A1** | **ApoA1** | **ApoB** | **ApoB/A1** |
| Vegetables | 0.042** | -0.018 | -0.042** | 0.032* | -0.013 | -0.031* | N.S. | N.S. | -0.021* |
| Fruit and berries | -0.045** | -0.013 | 0.008 | -0.016 | -0.003 | 0.001 | -0.024* | N.S. | N.S. |
| Potatoes | -0.028* | 0.016 | 0.032* | -0.020 | 0.010 | 0.022* | -0.036** | N.S. | N.S. |
| Grain/Cereals <15% sugar | -0.049** | -0.075** | -0.035** | -0.045** | -0.046** | -0.014 | -0.036** | -0.047** | N.S. |
| Grain/Cereals >15% sugar | -0.017 | -0.019 | -0.009 | -0.028* | -0.004 | 0.009 | -0.034* | N.S. | N.S. |
| Low-fibre bread | -0.057** | -0.036** | 0.006 | -0.023* | -0.024* | -0.005 | -0.039** | -0.025* | N.S. |
| High-fibre bread | 0.031* | -0.018* | -0.036** | 0.016 | -0.004 | -0.014 | N.S. | N.S. | N.S. |
| Rice and pasta | -0.003 | -0.029* | -0.025* | -0.002 | -0.017 | -0.015 | N.S. | N.S. | N.S. |
| Pastries | -0.078** | -0.047** | 0.003 | -0.064** | -0.018 | 0.018 | -0.062** | N.S. | N.S. |
| Egg | 0.033** | 0.008 | -0.014 | 0.034** | -0.012 | -0.030* | 0.027* | N.S. | -0.031* |
| Non-processed meat | 0.009 | 0.062** | 0.044** | 0.002 | 0.039** | 0.029* | N.S. | 0.034* | 0.033* |
| Processed meat | 0.019 | 0.034** | 0.014 | 0.039** | 0.015 | -0.013 | 0.029* | N.S. | N.S. |
| Poultry | 0.025* | 0.016 | -0.002 | 0.013 | 0.008 | -0.001 | N.S. | N.S. | N.S. |
| Fish and shellfish | 0.058** | 0.014 | -0.020* | 0.024* | 0.009 | -0.003 | N.S. | N.S. | N.S. |
| Fermented milk | 0.007 | -0.033** | -0.031* | -0.003 | -0.013 | -0.009 | N.S. | N.S. | N.S. |
| Non-fermented milk | -0.110** | 0.018 | 0.078** | -0.034** | 0.010 | 0.028* | -0.029* | N.S. | 0.024* |
| Cream | 0.039** | 0.010 | -0.023* | 0.009 | 0.019 | 0.002 | N.S. | N.S. | N.S. |
| Cheese | 0.011 | -0.010 | -0.021* | -0.003 | -0.007 | -0.009 | N.S. | N.S. | N.S. |
| Ice cream | -0.056** | -0.025* | 0.005 | -0.045** | -0.018 | 0.005 | -0.039** | -0.023* | N.S. |
| Margarine | -0.037** | -0.010 | 0.011 | -0.018 | -0.009 | 0.001 | N.S. | N.S. | N.S. |
| Butter-based fat | 0.055** | 0.012 | -0.020 | 0.043** | 0.015 | -0.012 | 0.033* | N.S. | N.S. |
| Oil/mayo/dressing | 0.044** | -0.006 | -0.029* | 0.008 | -0.008 | -0.009 | N.S. | N.S. | N.S. |
| Sweets | -0.028* | 0.040** | 0.049** | -0.023* | 0.032* | 0.040** | N.S. | 0.034* | 0.036** |
| Chocolate | -0.020 | 0.010 | 0.016 | -.022 | 0.019 | 0.026* | N.S. | N.S. | N.S. |
| Snacks/nuts | 0.043** | 0.031* | -0.006 | -0.003 | 0.023* | 0.014 | N.S. | N.S. | N.S. |
| Jam/Sugar | -0.090** | -0.026* | 0.032* | -0.070** | 0.004 | 0.043** | -0.058** | N.S. | 0.041** |
| Fruit juice | 0.016 | -0.001 | -0.015 | -0.004 | 0.009 | 0.005 | N.S. | N.S. | N.S. |
| Coffee | -0.022* | 0.031* | 0.036** | 0.002 | 0.001 | -0.005 | N.S. | N.S. | N.S. |
| Tea | -0.016 | -0.048** | -0.031* | -0.030* | -0.018 | 0.003 | -0.027* | N.S. | N.S. |
| Sugar-sweetened beverages | -0.064** | 0.010 | 0.044** | -0.042** | 0.001 | 0.024* | -0.038** | N.S. | 0.021* |
| Artificially-sweetened beverages | -0.013 | 0.006 | 0.010 | 0.007 | -0.010 | -0.013 | N.S. | N.S. | N.S. |
| Beer | 0.168** | 0.010 | -0.088** | 0.166** | 0.013 | -0.086** | 0.085** | N.S. | -0.056** |
| Wine | 0.191** | 0.040** | -0.079** | 0.190** | 0.051** | -0.070** | 0.118** | 0.036* | -0.042** |
| Spirits | 0.143** | 0.062** | -0.033* | 0.162** | 0.040** | -0.064** | 0.068** | N.S. | -0.025* |

*P-value between 0.05 and 0.0015 (nominal significance)

**P-value <0.0015 (Bonferroni-corrected significance)

1: Basic model adjusted for age, screening date and total energy intake.

2: Multivariable model adjusted for age, screening date, total energy intake, BMI, leisure-time physical activity, alcohol and smoking habits. The analyses with beer, wine and spirit were not adjusted for alcohol.

3: Stepwise backward elimination linear regression analysis, in which all food variables were simultaneously included. The model was adjusted for age, screening date, total energy intake, BMI, leisure-time physical activity, and smoking habits. N.S. indicate an eliminated variable with p >0.10.

**Table S3.** Partial correlation coefficients between dietary intake and the serum apolipoprotein concentrations among women in the Malmö Diet and Cancer cohort (1991–1996)

|  | **Basic multivariable model^1^** | | | **Full multivariable model^2^** | | | **Mutually adjusted model^3^** | | |
| --- | --- | --- | --- | --- | --- | --- | --- | --- | --- |
| **Food group** | **ApoA1** | **ApoB** | **ApoB/A1** | **ApoA1** | **ApoB** | **ApoB/A1** | **ApoA1** | **ApoB** | **ApoB/A1** |
| Vegetables | 0.038** | -0.025** | -0.041** | 0.010 | -0.008 | -0.013 | N.S. | N.S. | N.S. |
| Fruit and berries | -0.004 | -0.022* | -0.018* | -0.007 | -0.014 | -0.009 | N.S. | N.S. | N.S. |
| Potatoes | -0.027** | 0.051** | 0.055** | -0.004 | 0.031** | 0.028** | N.S. | 0.026* | 0.026* |
| Grain/cereals <15% sugar | -0.023* | -0.043** | -0.023* | -0.028** | -0.029** | -0.009 | -0.020* | -0.025* | N.S. |
| Grain/cereals >15% sugar | 0.015 | -0.035** | -0.037** | -0.005 | -0.017* | -0.012 | N.S. | -0.016* | N.S. |
| Low fibre bread | -0.049** | 0.018* | 0.041** | -0.015 | 0.007 | 0.015 | -0.018* | N.S. | N.S. |
| High fibre bread | 0.038** | -0.023* | -0.040** | 0.014 | -0.008 | -0.016* | N.S. | N.S. | N.S. |
| Rice and pasta | -0.002 | -0.021 | -0.019* | -0.011 | -0.008 | -0.004 | N.S. | N.S. | N.S. |
| Pastries | -0.030** | 0.009 | 0.019* | -0.022* | 0.017* | 0.023* | N.S. | N.S. | N.S. |
| Egg | 0.002 | 0.013 | 0.009 | 0.010 | -0.003 | -0.008 | N.S. | N.S. | N.S. |
| Non-processed meat | -0.010 | 0.044** | 0.042** | -0.001 | 0.022* | 0.020* | N.S. | N.S. | N.S. |
| Processed meat | -0.020* | 0.043** | 0.046** | 0.013 | 0.014 | 0.007 | 0.021* | N.S. | N.S. |
| Poultry | 0.012 | 0.007 | 0.001 | 0.002 | 0.008 | 0.006 | N.S. | N.S. | N.S. |
| Fish and shellfish | 0.061** | 0.007 | -0.023* | 0.029** | 0.022* | 0.005 | 0.020* | 0.021* | N.S. |
| Fermented milk | 0.039** | -0.058** | -0.067** | 0.015 | -0.038** | -0.039** | N.S. | -0.025* | -0.032** |
| Non-fermented milk | -0.101** | 0.048** | 0.092** | -0.040** | 0.013 | 0.034** | -0.030** | 0.017* | 0.025* |
| Cream | 0.067** | -0.020* | -0.051** | 0.020* | 0.009 | -0.003 | 0.019* | N.S. | N.S. |
| Cheese | 0.066** | -0.048** | -0.073** | 0.044** | -0.034** | -0.052** | 0.037** | -0.026* | -0.038** |
| Ice cream | -0.007 | -0.029** | -0.022* | 0.001 | -0.032** | -0.028** | N.S. | -0.033** | -0.033** |
| Margarine | -0.043** | 0.019* | 0.037** | -0.018* | 0.001 | 0.009 | N.S. | N.S. | N.S. |
| Butter based fat | 0.045** | -0.010 | -0.031** | 0.030** | 0.003 | -0.013 | 0.028** | N.S. | -0.017* |
| Oil/mayo/dressing | 0.035** | -0.002 | -0.022* | 0.008 | 0.007 | -0.002 | N.S. | N.S. | N.S. |
| Sweets | -0.042** | 0.048** | 0.061** | -0.027** | 0.030** | 0.040** | -0.021* | 0.029** | 0.036** |
| Chocolate | -0.012 | 0.018* | 0.017* | -0.022* | 0.022* | 0.026** | -0.018* | 0.019* | 0.022* |
| Snacks/nuts | 0.024* | -0.005 | -0.018* | -0.008 | 0.004 | 0.005 | N.S. | N.S. | N.S. |
| Jam/sugar | -0.041** | 0.008 | 0.028** | -0.031** | 0.017* | 0.032** | -0.016* | N.S. | 0.020* |
| Fruit juice | 0.010 | -0.005 | -0.011 | -0.009 | 0.010 | 0.011 | N.S. | N.S. | N.S. |
| Coffee | -0.023* | 0.016* | 0.027** | -0.004 | -0.013 | -0.008 | N.S. | N.S. | N.S. |
| Tea | 0.022* | -0.045** | -0.050** | -0.006 | -0.014 | -0.011 | N.S. | N.S. | N.S. |
| Sugar-sweetened beverages | -0.077** | 0.063** | 0.088** | -0.050** | 0.046** | 0.061** | -0.045** | 0.044** | 0.057** |
| Artificially-sweetened beverages | -0.040** | 0.032** | 0.046** | -0.017* | 0.006 | 0.014 | N.S. | N.S. | N.S. |
| Beer | 0.094** | -0.031** | -0.074** | 0.082** | -0.015 | -0.056** | 0.049** | N.S. | -0.036** |
| Wine | 0.195** | -0.070** | -0.154** | 0.174** | -0.045** | -0.127** | 0.125** | -0.046** | -0.096** |
| Spirits | 0.099** | -0.015 | -0.063** | 0.102** | -0.018* | -0.069** | 0.047** | N.S. | -0.033** |

*P-value between 0.05 and 0.0015 (nominal significance)

**P-value <0.0015 (Bonferroni-corrected significance)

1: Basic model adjusted for age, screening date and total energy intake.

2: Multivariable model adjusted for age, screening date, total energy intake, BMI, leisure-time physical activity, alcohol and smoking habits. The analyses including beer, wine and spirit were not adjusted for alcohol.

3: Stepwise backward elimination linear regression analysis, in which all food variables simultaneously included. The model was adjusted for age, screening date, total energy intake, BMI, leisure-time physical activity, and smoking habits. N.S. indicate an eliminated variable with p >0.10.

**Table S4.** Partial correlation coefficients between lifestyle factors, dietary intake and serum HDL-C and LDL-C concentrations among 1844 men and 2809 women in the Malmö Diet and Cancer cohort (1991–1994)

|  | **Men**  **Full multivariable model^1^** | | | **Women**  **Full multivariable model^1^** | | |
| --- | --- | --- | --- | --- | --- | --- |
| **Variables** | **HDL-C** | **LDL-C** | **LDL-C/HDL-C** | **HDL-C** | **LDL-C** | **LDL-C/HDL-C** |
| Alcohol habits | 0.222 | 0.045 | -0.133 | 0.180 | -0.044 | -0.145 |
| BMI | -0.301 | 0.059 | 0.233 | -0.285 | 0.094 | 0.249 |
| Education | 0.003 | 0.013 | -0.001 | 0.060 | -0.043 | -0.059 |
| Physical activity | 0.051 | -0.009 | -0.039 | 0.109 | 0.019 | -0.054 |
| Smoking habits | 0.084 | -0.025 | -0.085 | 0.089 | -0.028 | -0.087 |
| Carbohydrates | -0.092 | -0.062 | 0.012 | -0.079 | 0.010 | 0.056 |
| Sucrose | -0.122 | -0.008 | 0.075 | -0.086 | 0.024 | 0.071 |
| Fiber | 0.005 | -0.011 | -0.022 | 0.020 | -0.006 | -0.018 |
| Protein | 0.086 | 0.019 | -0.053 | 0.046 | -0.019 | -0.042 |
| Fat | 0.059 | 0.068 | 0.015 | 0.060 | -0.010 | -0.044 |
| SFA | 0.030 | 0.040 | 0.018 | 0.066 | -0.004 | -0.044 |
| MUFA | 0.066 | 0.062 | 0.008 | 0.038 | -0.001 | -0.022 |
| PUFA | 0.045 | 0.036 | -0.011 | 0.001 | -0.007 | -0.006 |
| Omega-3 PUFA | 0.095 | 0.067 | -0.014 | 0.050 | 0.002 | -0.021 |
| Omega-6 PUFA | 0.021 | 0.021 | -0.005 | -0.009 | -0.005 | -0.002 |
| Vegetables | 0.057 | -0.002 | -0.031 | 0.035 | 0.012 | -0.017 |
| Fruit and berries | -0.021 | -0.037 | -0.018 | 0.032 | 0.011 | -0.012 |
| Potatoes | -0.039 | 0.010 | 0.033 | -0.015 | 0.002 | 0.012 |
| Grain/cereals <15% sugar | -0.032 | -0.029 | 0.001 | -0.019 | -0.024 | -0.011 |
| Grain/cereals >15% sugar | -0.015 | -0.003 | -0.002 | -0.001 | -0.035 | -0.024 |
| Low-fibre bread | -0.038 | -0.027 | -0.001 | -0.008 | -0.007 | 0.006 |
| High-fibre bread | 0.053 | 0.034 | -0.023 | -0.002 | 0.020 | 0.009 |
| Rice and pasta | 0.005 | -0.013 | -0.002 | 0.016 | -0.010 | -0.028 |
| Pastries | -0.099 | 0.023 | 0.072 | -0.027 | 0.042 | 0.049 |
| Egg | 0.035 | -0.028 | -0.036 | 0.013 | -0.009 | -0.019 |
| Non-processed meat | 0.025 | 0.054 | 0.023 | -0.029 | 0.004 | 0.019 |
| Processed meat | 0.034 | -0.010 | -0.035 | -0.015 | -0.005 | 0.019 |
| Poultry | 0.023 | 0.028 | 0.003 | -0.003 | 0.031 | 0.021 |
| Fish and shellfish | 0.062 | 0.059 | -0.013 | 0.040 | 0.033 | 0.012 |
| Fermented milk | 0.021 | -0.036 | -0.034 | 0.026 | -0.001 | -0.025 |
| Non-fermented milk | -0.028 | -0.014 | 0.002 | -0.048 | 0.021 | 0.043 |
| Cream | 0.026 | 0.052 | 0.018 | 0.008 | 0.046 | 0.026 |
| Cheese | 0.001 | 0.011 | 0.002 | 0.073 | -0.053 | -0.087 |
| Ice cream | -0.021 | 0.047 | 0.047 | 0.015 | -0.015 | -0.020 |
| Margarine | -0.020 | -0.009 | -0.004 | -0.020 | -0.019 | -0.008 |
| Butter based fat | 0.009 | 0.028 | 0.016 | 0.035 | 0.005 | -0.016 |
| Oil/mayo/dressing | 0.030 | 0.009 | -0.009 | -0.017 | -0.009 | 0.002 |
| Sweets | -0.034 | 0.045 | 0.049 | -0.029 | 0.050 | 0.060 |
| Chocolate | -0.066 | 0.020 | 0.060 | -0.022 | 0.031 | 0.034 |
| Snacks/nuts | 0.010 | 0.062 | 0.038 | -0.018 | 0.008 | 0.013 |
| Jam/sugar | -0.078 | -0.026 | 0.040 | -0.058 | -0.024 | 0.026 |
| Fruit juice | -0.009 | -0.029 | -0.018 | 0.011 | 0.023 | 0.003 |
| Coffee | 0.018 | 0.034 | -0.004 | 0.043 | 0.024 | -0.018 |
| Tea | 0.005 | -0.033 | -0.031 | 0.014 | -0.027 | -0.023 |
| Sugar-sweetened beverages | -0.086 | 0.007 | 0.070 | -0.056 | 0.031 | 0.056 |
| Artificially-sweetened beverages | 0.001 | -0.012 | -0.016 | -0.004 | -0.011 | -0.011 |
| Beer | 0.169 | 0.026 | -0.111 | 0.074 | -0.008 | -0.063 |
| Wine | 0.202 | 0.060 | -0.098 | 0.153 | -0.026 | -0.110 |
| Spirits | 0.142 | 0.049 | -0.068 | 0.098 | -0.024 | -0.080 |

1: Multivariable model adjusted for age, screening date, BMI, leisure-time physical activity, alcohol consumption and smoking habits. Dietary factors were also adjusted for total energy intake. The analyses including beer, wine and spirit were not adjusted for alcohol.
